# Case Report: Parapharyngeal Leiomyosarcoma Mimicking Neurofibroma

**DOI:** 10.1002/ccr3.72270

**Published:** 2026-03-08

**Authors:** Carson Brantley, Claudia N. Gutierrez, Sara Zadeh, Eric Dowling

**Affiliations:** ^1^ College of Arts & Sciences, University of Virginia Virginia USA; ^2^ Department of Otolaryngology – Head and Neck Surgery University of Virginia Virginia USA; ^3^ Department of Pathology and Laboratory Medicine University of Virginia Virginia USA

**Keywords:** head and neck neoplasms, leiomyosarcoma, neurofibroma, otolaryngology, parapharyngeal space

## Abstract

Leiomyosarcomas and neurofibromas of the parapharyngeal space share similar clinical and radiographic features, making early diagnosis challenging. In patients without neurocutaneous disorders, an adequate tissue sample with immunohistochemistry is crucial for diagnosis. Early detection and treatment of leiomyosarcoma are essential to improve survival and prevent progression of disease.

## Graphical abstract

Neurofibroma and leiomyosarcomas are rare tumors of the head and neck with similar imaging characteristics of T2 hyperintensity on MRI that can present with neurological deficits. However, leiomyosarcomas are malignant, and early diagnosis is critical to improved survival. Immunohistochemistry is crucial for assigning line of differentiation in spindle cell tumors, especially on limited samples.

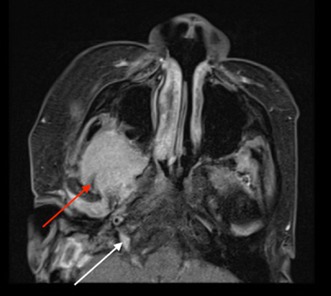

## 1 Introduction

Parapharyngeal space tumors are uncommon, accounting for 0.5%–1.5% of all head and neck tumors, with the majority being benign (71%). The most common benign tumor is pleomorphic adenoma, while squamous cell carcinoma is the most frequent malignancy. These tumors are often asymptomatic but can present with dysphagia, dyspnea, dysphonia, neck swelling, or pain [1].

Leiomyosarcomas are malignant smooth muscle tumors that rarely occur in the head and neck region, with only five previously reported cases of primary parapharyngeal space involvement. Neurofibromas, on the other hand, are benign peripheral nerve sheath tumors that can arise sporadically or in association with neurofibromatosis type 1 (NF1).

Differentiating between leiomyosarcomas and neurofibromas of the parapharyngeal space can be challenging due to their similar clinical presentations and radiographic features [1]. Morphology and immunohistochemistry play a crucial role in the classification of these tumors. For example, leiomyosarcomas frequently show greater cytologic atypia and an immunoprofile positive for smooth muscle actin (SMA) and desmin but negative for S‐100 expression. Conversely, neurofibromas show minimal atypia and are positive for S‐100 but negative for SMA and desmin [2].

We report the sixth case of primary leiomyosarcoma of the parapharyngeal space and discuss the diagnostic challenges and importance of early detection for improved survival.

## 2 Case History/Examination

**FIGURE 1:**
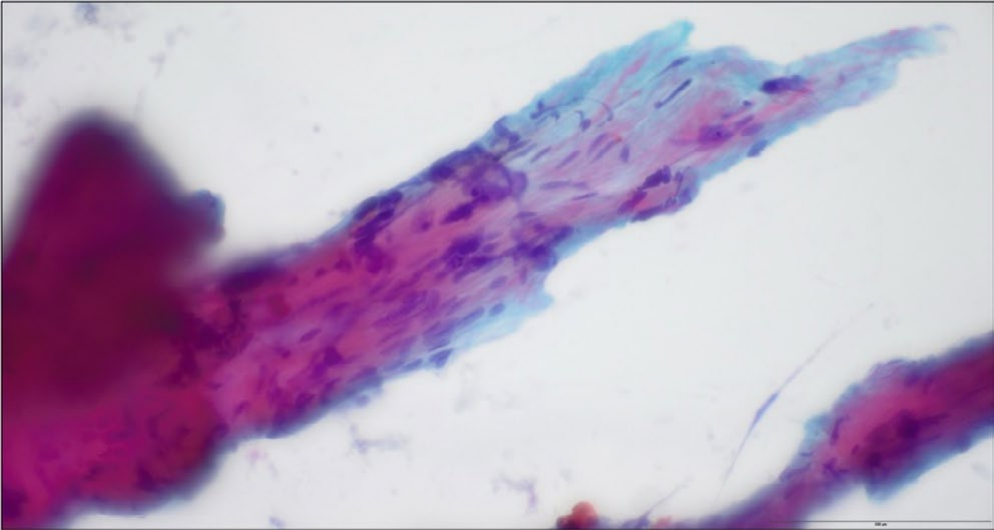
Preoperative fine‐needle aspiration demonstrating scant material and rare fragments of bland spindled cells. The accompanying cell block material was acellular.

A 55‐year‐old woman presented with progressive hoarseness, right otalgia, and dysphonia. Flexible nasolaryngoscopy identified a right true vocal fold paralysis and a CT revealed a 1.8 × 3.3 × 2.0 cm right carotid space mass, suspicious for a neurofibroma. CT‐guided fine‐needle aspiration (FNA) biopsy showed bland spindle cells, which raised suspicion for a peripheral nerve sheath tumor; however, the sample was small and had scant cellularity (Figure 1). The patient initially declined surgery of this suspected neurofibroma due to socioeconomic reasons and concerns about the procedure. Seven months after this initial biopsy, she developed palatal asymmetry, nasopharyngeal regurgitation, ptosis, and hypoglossal nerve dysfunction.

## 3 Methods

**FIGURE 2:**
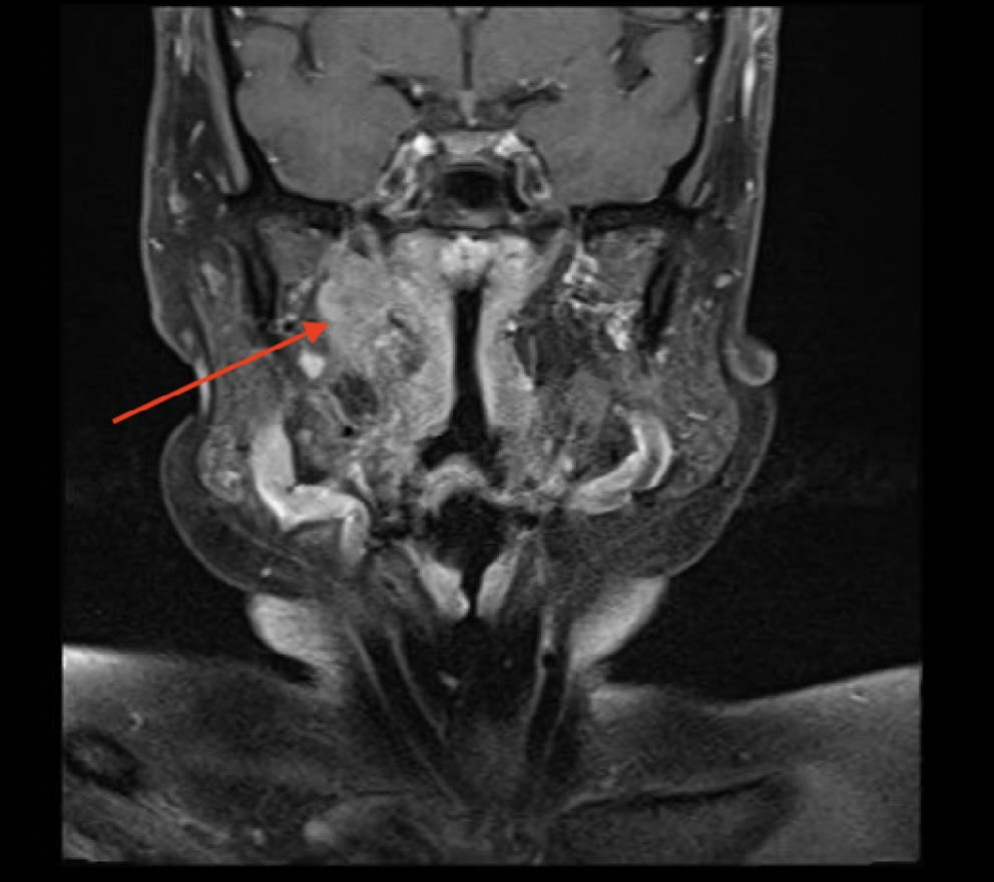
Preoperative T2‐weighted coronal MRI indicating an enhancing mass of the right parapharyngeal space (red arrow) with anteromedial displacement of the hypoglossal nerve and posterolateral displacement of the internal carotid artery.

**FIGURE 3:**
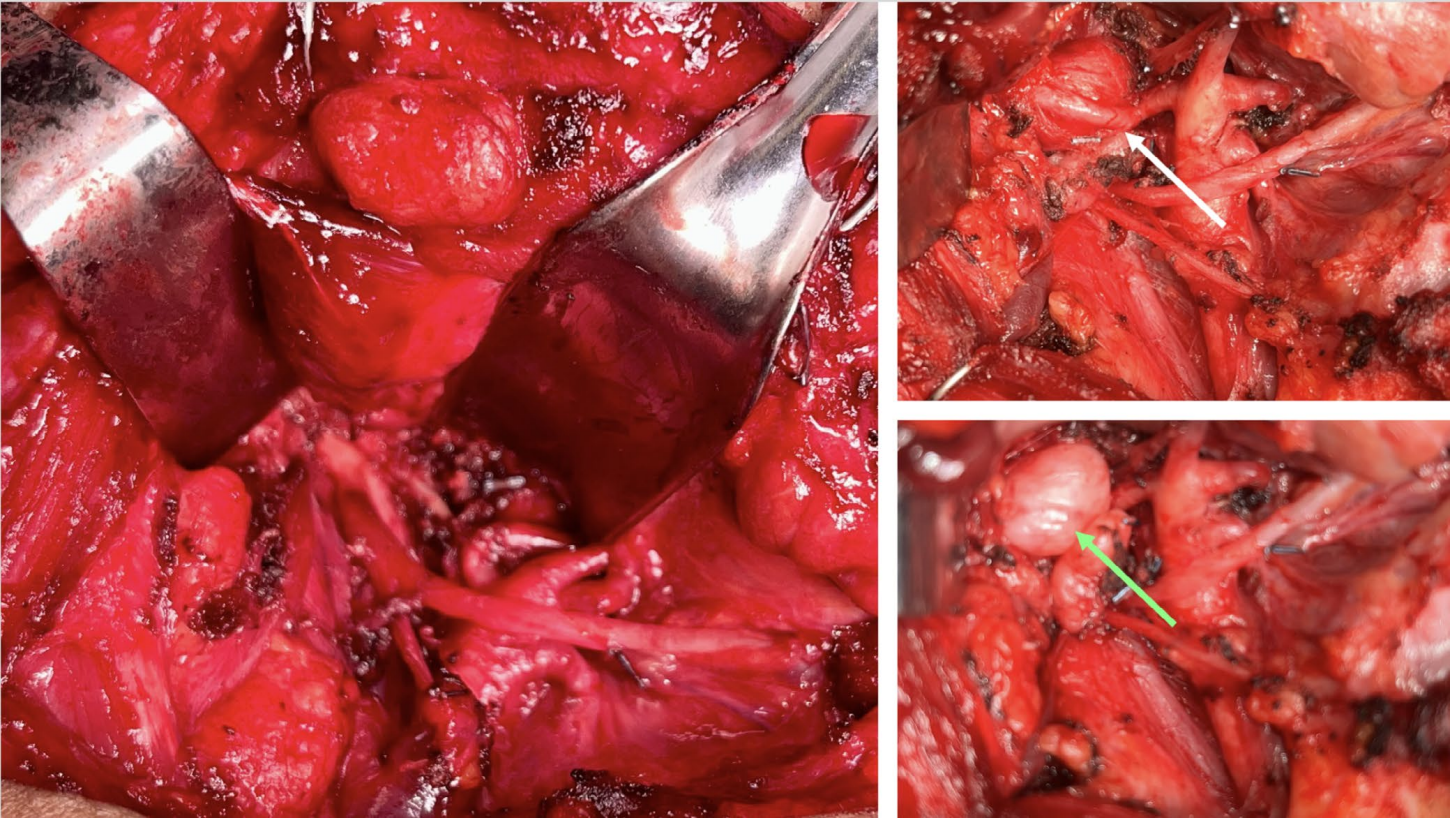
Intraoperative images of the parapharyngeal tumor. Left: Resection bed after tumor extirpation. Right, top: Occipital artery crossing superficial to tumor (white arrow). Right, bottom: Tumor (green arrow) overlying hypoglossal nerve at the skull base after ligation of occipital artery.

In this 7‐month follow‐up, resection was again recommended given the interval growth of the mass and new cranial neuropathies. MRI showed a slightly enlarged, 2.1 × 3.3 × 2.0 cm, T2 hyperintense mass with possible jugular vein invasion and denervation changes of the right tongue (Figure 2). This case was presented at the multidisciplinary tumor board where the possibility of performing a mandibulotomy was discussed but was deferred in favor of a less invasive alternative due to the suspected benign nature of the lesion, lack of clear intracranial or intraforaminal extension, and the patient's preference to avoid a morbid exposure. The patient underwent transmastoid occlusion of the sigmoid sinus, transcervical approach to the right parapharyngeal space for tumor excision, and right laryngeal reinnervation using right ansa cervicalis (Figure 3). The tumor was densely adherent to the hypoglossal nerve at the skull base but was excised with gross negative margins.

**FIGURE 4:**
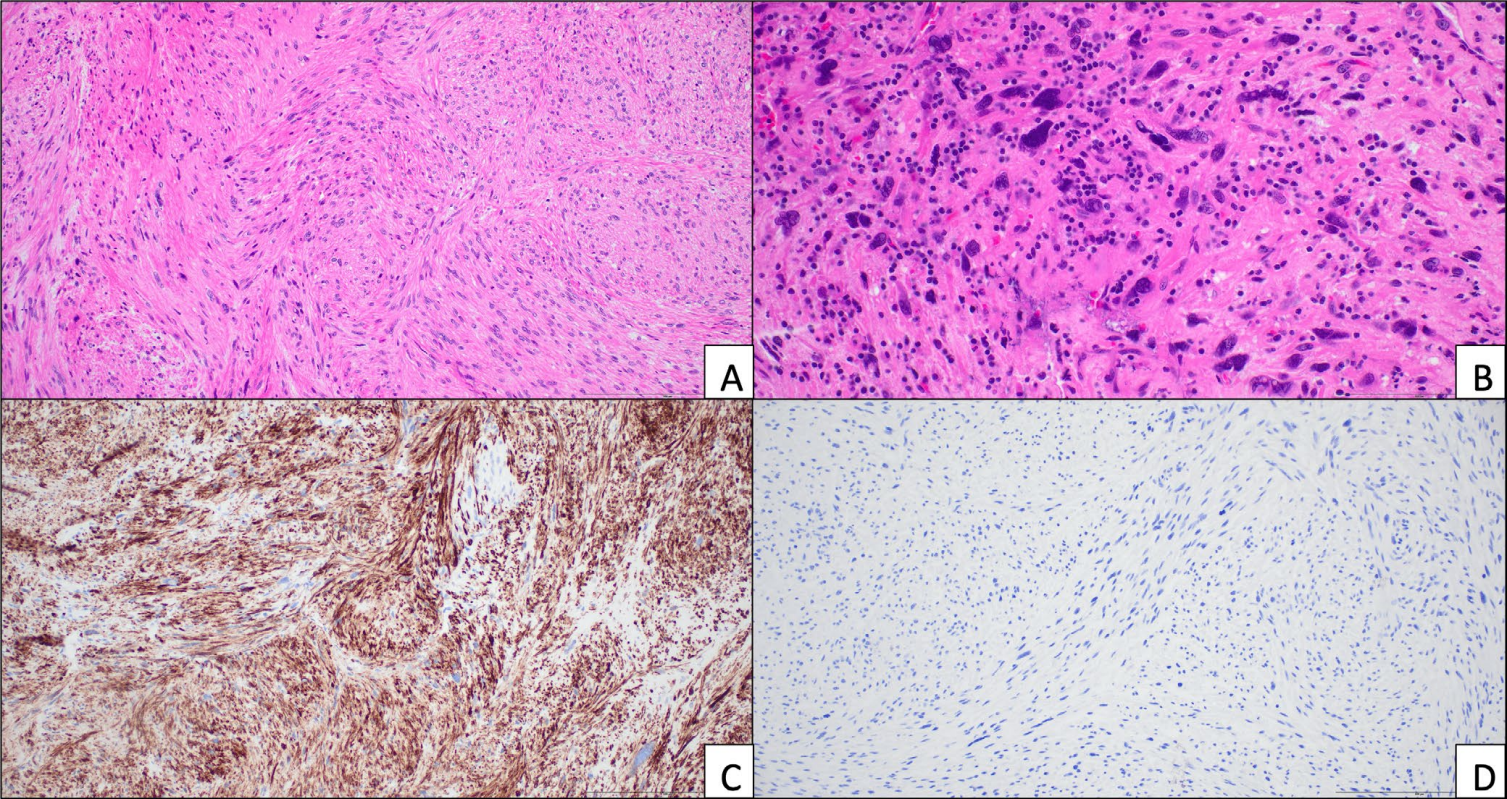
Resection specimen pathology. (A) H&E section showing spindled cells and mild cytologic atypia similar to the biopsy. (B) Other areas show significant pleomorphism with large nuclei and hyperchromasia. (C) Immunohistochemical stain for desmin was strongly, diffusely positive, indicating smooth muscle differentiation. (D) Stain for S‐100 was negative, providing no support for nerve sheath differentiation and neurofibroma.

Final pathology on this larger sample revealed a cellular spindle cell neoplasm with significant cytologic atypia. Immunohistochemistry was positive for desmin and SMA but negative for S‐100, supporting smooth muscle differentiation (Figure 4). The specimen showed an increased Ki‐67 proliferation index and a mitotic index of 0–5 mitoses per 10 high‐power fields. Taken together, the findings from this intraoperative specimen taken 7 months after the initial biopsy were that of a grade 2 leiomyosarcoma rather than the initially suspected neurofibroma.

## 4 Conclusion and Results

**FIGURE 5:**
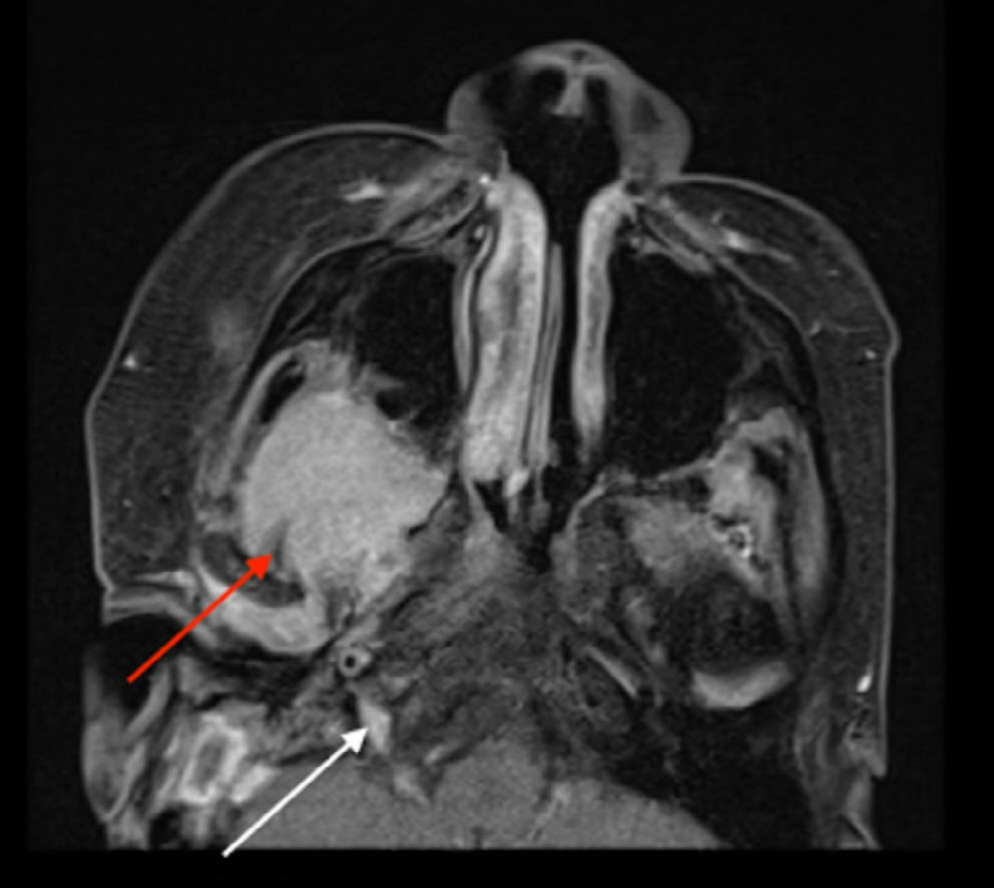
Two‐month postoperative MRI with red arrow indicating recurrence advancing into the right infratemporal fossa (red arrow, changed from previous imaging) with extension through the right hypoglossal canal in close proximity to the right Meckel's cave (white arrow).

A 2‐month postoperative MRI revealed a recurrence of the tumor centered in the right infratemporal fossa (Figure 5). The patient underwent PEG placement due to dysphagia and underwent palliative radiotherapy. She completed 60 Gy in 30 fractions of radiotherapy to the operative bed with plans to start palliative chemotherapy due to the discovery of hepatic, pulmonary, and osseous metastases; however, her symptoms worsened and she transitioned to hospice. She passed 14 months after her initial biopsy and 7 months after her formal diagnosis.

## 5 Discussion

Leiomyosarcomas and neurofibromas of the parapharyngeal space present with similar clinical and radiographic features, making early diagnosis challenging. In patients without a history of neurocutaneous disorders, an adequate tissue sample with immunohistochemistry is crucial for definitive diagnosis of spindle cell neoplasms. Early detection and treatment are essential for improved survival in leiomyosarcoma cases, as delayed management can lead to advanced stage at diagnosis and worse oncologic outcomes. Both entities can present with dysphagia, dyspnea, dysarthria, neck swelling, or pain. On imaging, both tumors appear as well‐defined, T2 hyperintense masses on MRI [1].

Cranial neuropathies, although more commonly associated with malignant lesions, can occur in both leiomyosarcomas and neurofibromas [1, 3]. In a systematic review of 1143 parapharyngeal space tumors, cranial neuropathy was the third most common sign. Cranial neuropathies were reported in 18% (*n* = 95) of cases with the vagus nerve being the most frequently affected [3].

Fine‐needle aspiration biopsy may not provide sufficient cellular material for definitive diagnosis, as seen in this case. Core needle biopsy could potentially offer more conclusive results but carries risks due to the complex anatomy of the parapharyngeal space [1]. In a study by Chen et al., core needle biopsy of parapharyngeal space lesions had a diagnostic accuracy of 90.9%, with no major complications reported [4].

Immunohistochemistry is crucial for assigning line of differentiation in spindle cell tumors, especially on limited samples. Leiomyosarcomas are malignant tumors that show smooth muscle differentiation (positive SMA and desmin, negative S‐100). Neurofibromas are benign peripheral nerve sheath tumors (positive S‐100, negative SMA and desmin) [2]. Leiomyosarcomas typically have greater cytologic atypia, increased mitotic activity, and may have necrosis compared to benign smooth muscle tumors [2]. Given that the initial FNA sample had limited tumor cells, an immunopanel was not able to be performed which limited definitive diagnosis. This emphasizes the importance of an adequate pathology sample with the opportunity to employ immunohistochemistry to further classify spindled soft tissue neoplasms.

Early diagnosis and treatment are essential for improved survival in leiomyosarcoma cases, as delayed management can lead to worsening neurological deficits and poorer oncologic outcomes. In a review of 578 cases of head and neck leiomyosarcoma, Eppsteiner et al. reported a 5‐year survival rate of 87.6% for well‐differentiated tumors and 52.7% for poorly differentiated tumors, with tumor size and grade being significant prognostic factors [5].

When sarcoma is suspected, more aggressive surgical intervention, such as mandibulotomy with wider field extirpation, may be warranted. In this case, discussion at the multidisciplinary tumor board resulted in a less invasive surgical approach due to recommendation from neuroradiology and the patient's preference. Although a surgical approach was performed seeking to provide safe skull base venous control, the sarcomatous biology of the mass declared itself on recurrence. At that time, the patient presented with multiple distant metastases and opted for palliative care.

This case highlights the importance of a multidisciplinary approach in managing parapharyngeal space tumors, allowing for tumor resection and functional restoration. The complex anatomy of the parapharyngeal space necessitates careful preoperative planning and collaboration among otolaryngologists, medical oncologists, radiation oncologists, and pathologists to achieve optimal outcomes.

## Author Contributions

**Carson Brantley:** conceptualization, formal analysis, writing – original draft, writing – review and editing. **Claudia N. Gutierrez:** conceptualization, methodology, supervision, writing – original draft, writing – review and editing. **Sara Zadeh:** data curation, formal analysis, methodology, validation, writing – review and editing. **Eric Dowling:** conceptualization, data curation, formal analysis, investigation, methodology, project administration, writing – review and editing.

## Funding

The authors have nothing to report.

## Consent

Written informed consent was obtained from the patient's next of kin to publish this report in accordance with the journal's patient consent policy.

## Conflicts of Interest

The authors declare no conflicts of interest.

## Data Availability

Data sharing is not applicable to this article as this is a retrospective review of a single case.
